# Supplementary material for: Reference intervals of common clinical biochemistry analytes in young Nigerian adults
Source: PLoS One. 2021 Mar 1;16(3):e0247672. doi: 10.1371/journal.pone.0247672 (PMC7920356; doi:10.1371/journal.pone.0247672)
Supplement: S1 File — (DOCX) [file pone.0247672.s001.docx]

**Authors’ List / Contributions**

1. ***Dr Ojor Ayemoba** - (Corresponding Author)

Highest Academic Degree: FMCPath (Haematology)

Phone: +2348023098494

Email: [orayemoba@yahoo.com](mailto:orayemoba@yahoo.com)

ORCID No: https://orcid.org/0000-0003-2443-0323

2. **Dr Nathan Okeji**

Highest Academic Degree: FWACS

Phone: +2348033438417

Email: [nokeji@yahoo.com](mailto:nokeji@yahoo.com)

ORCID No. <https://orcid.org/>0000-0001-6430-225X

3. **Dr Nurudeen Hussain**

Highest Academic Degree: FWACP

Phone: +2348033921922

Email: [naahussain@yahoo.co.uk](mailto:naahussain@yahoo.co.uk)

ORCID No: https://orcid.org/0000-0002-1419-2467

4. **Dr Tahir Umar**

Highest Academic Degree: FWACP

Phone +2348038249415

Email: [tahiroshmar@gmail.com](mailto:tahiroshmar@gmail.com)

ORCID No: https://orcid.org/0000-0002-2303-6191

5. **Dr Anthony Ajemba-Life**

Highest Academic Degree: FWACP

Phone: +2348033781290

Email: [ajembalife@yahoo.com](mailto:ajembalife@yahoo.com)

ORCID No: https://orcid.org/0000-0001-9484-0665

6. **Uchechukwu Edom**

Highest Academic Degree: MSc (Public Health)

Phone: +2348037269128

Email: [edomite2002@yahoo.com](mailto:edomite2002@yahoo.com)

ORCID No: https://orcid.org/0000-0002-1491-8264

7. **Ikechukwu Ogueri**

Highest Academic Degree: MSc (Immunology)

Phone: +2348033226460

Email: [oguerii@yahoo.com](mailto:oguerii@yahoo.com)

ORCID No: https://orcid.org/0000-0002-7664-0165

8. **Goodluck Nwagbara**

Highest Academic Degree: MSc (Clinical Chemistry)

Phone: +2348033303766

Email: [goodiesalosteves@yahoo.co.uk](mailto:goodiesalosteves@yahoo.co.uk)

ORCID No: https://orcid.org/0000-0003-0301-1584

9. **Inalegwu Ochai**

Highest Academic Degree: MSc (Microbiology)

Phone: +2349091216168

Email: [ochaiinaj@yahoo.com](mailto:ochaiinaj@yahoo.com)

ORCID No: https://orcid.org/0000-0001-7320-3237

10. **Usman Adekanye**

Highest Academic Degree: MPH (Field Epidemiology)

Phone: +2348068576712

Email: [adekanyeusmanoladipo@gmail.com](mailto:adekanyeusmanoladipo@gmail.com)

ORCID No: <https://orcid.org/0000-0003-1876-8598>

11. **Dr Terfa Kene**

Highest Academic Degree: FWACP

Ave Health Sense Limited

4 Balanga Street, Area 11,Abuja – Nigeria

Phone: +2348037053248

Email: [teskene@yahoo.com](mailto:teskene@yahoo.com)

ORCID No:<https://orcid.org/>0000-0002-2110-2407

12. **Ikenna Onoh**

Highest Academic Degree: FWACP

Nigerian Field Epidemiology and Laboratory Training Programme (FELTP)

50 Haile Selassie Street, Asokoro Abuja, Nigeria

Phone: +2348036354733

Email: [ikeonoh@yahoo.com](mailto:ikeonoh@yahoo.com)

ORCID No: https://orcid.org/0000-0002-2039-7215

**Authors’ Contributions**

O.R Ayemoba (MODHIP), N.A.A Hussain (MODHIP), T.O Umar (MODHIP) and A.E Ajemba-Life (MODHIP) were responsible for Protocol development, IRB approval, field supervision and manuscript writing. N.A.E. Okeji (MODHIP) provided administrative overview and participated in manuscript writing. U.S Edom (MODHIP), I. Ogueri (MODHIP), G.O.N Nwagbara (MODHIP) and I. Ochai (MODHIP) performed field data gathering, laboratory analysis and quality assurance activities. U. Adekanye (MODHIP) and I. Onoh (FELTP) were responsible for data capture, quality, storage, analysis and manuscript writing, while T.S Kene (Ave Health Sense Ltd, Abuja) participated in Protocol development, bio-statistical analysis and manuscript writing.
