# Supplementary material for: Reference intervals of common clinical biochemistry analytes in young Nigerian adults
Source: PLoS One. 2021 Mar 1;16(3):e0247672. doi: 10.1371/journal.pone.0247672 (PMC7920356; doi:10.1371/journal.pone.0247672)
Supplement: S3 File — (PDF) [file pone.0247672.s003.pdf]

CERTIFICATE OF ETHICS APPROVAL

M.O.D. Health Research Ethics Committee
Ship House, Area 10, Garki, Abuja.
Email: modhrec@gmail.com. Tel: 0802 309 3714

MODHREC/APP/ 037
24th Jan. 2014.

TAHIR UMAR (Maj Gen), MB; BS., FWACP

Emergency Plan Implementation Committee Liaison office
Nigeria Ministry of Defence
4B, Ikole street Area 11 Garki Abuja.

NOTICE OF REVIEW AND APPROVAL OF PROTOCOL TITLED *PREVALENCE OF HIV, HEPATITIS-B AND DETERMINATION OF SOME HEAMATOLOGICAL AND BIOCHEMICAL REFERENCE VALUES AMONG NIGERIA MILITARY SERVICE APPLICANTS.*

This is to inform you that your request for approval of the research described in the submitted protocol mentioned above dated 24th December 2013 has been approved.

2. The approval is from 24th Jan 2014 to 23rd Jan 2015.

3. Kindly note that the **National Code of Health Ethics** requires you to comply with all institutional guidelines, rules and regulations, and with the tenets of the code, including ensuring that all adverse events are reported promptly to MODHREC. No changes are permitted in the research protocol without prior approval by the MODHREC, except in circumstances outlined in the National Code.

4. The MODHREC reserves the right to conduct compliance visits to your research site(s) without prior notification.

5. At the end of this study, a detailed report should be submitted to MODHREC office.

Dr. J. N. Ugwuegede
Chairman
