# Supplementary material for: Reference intervals of common clinical biochemistry analytes in young Nigerian adults
Source: PLoS One. 2021 Mar 1;16(3):e0247672. doi: 10.1371/journal.pone.0247672 (PMC7920356; doi:10.1371/journal.pone.0247672)
Supplement: S1 Appendix — (DOCX) [file pone.0247672.s004.docx]

S1 APPENDIX

**CONSENT FORM**

**Ministry of Defence Emergency Plan Implementation Committee**

**CONSENT TO BE A STUDY PARTICIPANT**

TITLE: PREVALENCE OF HIV, HEPATITIS-B AND DETERMINATION OF SOME HAEMATOLOGICAL AND BIOCHEMICAL REFERENCE VALUES AMONG NIGERIAN MILITARY SERVICE APPLICANTS

**INTRODUCTION**

You are being asked to take part in this study because you are an applicant wishing to enlist into Nigerian military service. This study is paid for by MOD EPIC. Before you decide if you want to take part in this study, we want you to know about the study.

This is a consent form. It gives you information about this study. You will be given time to think about participating in this study before signing this consent form. You are free to ask questions about this study at any time. If you agree to take part in this study, you will be asked to sign this consent form and a copy will be given to you.

Please note that:

• Participation in this study is entirely voluntary.

• You are free to stop being part of the study at any time.

**WHY IS THIS STUDY BEING DONE?**

This study is being done:

- To determine the prevalence of HIV and Hepatitis-B among Nigerian military applicants
- To determine some hematological and biochemical reference values among Nigerians applying for military service
- To ensure that medically fit young people are recruited for service in the Nigerian military

**WHAT IS EXPECTED OF ME IN THIS STUDY?**

If you decide to take part in this study, you will complete an interview with the study staff where you will be asked questions about yourself. You will have some laboratory tests to screen you for HIV and Hepatitis-B. About 1 tablespoon (12.5mls) of blood will be taken for all of these tests. You may be contacted for care and treatment if the doctor has concern about your results. Any information collected from you will be treated with utmost privacy and confidentiality.

**HOW MANY PEOPLE WILL TAKE PART IN THIS STUDY?**

Each recruitment centre will enroll on average 400 applicants/state that are eligible and have qualified for medical assessment across the Federation including all eligible female applicants.

**INCLUSION CRITERIA**

- Application for enrollment into Nigerian military service
- Passed initial pre-assessment stage of recruitment exercise
- Willingness to participate in the study
- Male or Female applicant within the required age range (18-26years)
- Willingness and ability to provide informed consent
- Willingness to provide blood specimen

**EXCLUSION CRITERIA**

- Failed pre-assessment stage of recruitment exercise
- Inability to provide informed consent
- Unwillingness to participate in the study

**HOW LONG WILL I BE IN THIS STUDY?**

Your participation in this study will be for one day. You may however be referred to one of our service delivery points as may be indicated.

**REASONS FOR STUDY DISCONTINUATION**

You may be taken off this study at your own request or at the discretion of the study Principal Investigator.

**WHAT ARE THE RISKS IN THIS STUDY?**

There are very few risks to participating in the study. The study staff will ensure the reduction of all risks described below.

**Social Risks**

There is a small risk of having your HIV status revealed to others. If others learn you are HIV positive, it could cause you problems like stigmatization and discrimination. The study staff will take appropriate action to keep your information confidential and to assist you with any discrimination you may experience by being in this study.

**Blood Drawing Risks**

There is a slight chance that you may be harmed when blood is taken for your laboratory tests, just like for any other blood draw. These include discomfort, bleeding or bruising where the needle enters the body, light-headedness, and in rare cases fainting or infections.

**ARE THERE BENEFITS TO TAKING PART IN THIS STUDY?**

It is possible that you may receive no benefit from being in this study. By participating in this study, your doctor may learn more about your general well-being which may help you seek care. Although study participants may benefit from laboratory testing, they may receive no direct benefit from participating in the study. The data collected will help to improve the quality of medical care which may be of indirect benefit to study participants.

**WHAT IF I DON’T WANT TO PARTICIPATE IN THIS STUDY?**

Participation in this study is entirely voluntary.

**WHAT ABOUT CONFIDENTIALITY?**

The study team will provide you with a specific Study Identification Number (SIN). This identification number (not your name or other information that could be used to identify you directly) will be used for collecting all information from your clinical record and all your study records will be kept in a locked cabinet in a safe room. Only the study staff will have access to the room and cabinet. Further, any publication of this study will not use your name or identify you personally. Efforts will be made to keep your personal information confidential, but we cannot absolutely guarantee this and your personal information may be disclosed if required by law.

Your records from this research study may be reviewed by the MODHREC as part of their jobs to make sure that research subjects are protected.

**WILL I RECEIVE ANY PAYMENT?**

You will not be paid for participating in this study.

**WHAT ARE THE COSTS TO ME?**

You will not pay for any study visit, study procedure or laboratory tests.

**WHAT HAPPENS IF I AM INJURED?**

If you are injured as a result of being in this study, you will be provided free medical care for research-related injuries. There is no plan for compensation for illness or injury through the MOD program. You should discuss this thoroughly with the Principal Investigator or his designee before you enroll in this study. If you have any questions about any research-related injury, you may contact Dr. Tahir Oshe Umar (Major General) at telephone 08038249415. You should also understand that this is not a waiver or release of your legal rights.

**WHAT IS MY RIGHT AS A PARTICIPANT IN THIS STUDY?**

Taking part in this study is completely voluntary. You may choose not to take part in this study or leave this study at any time. You will receive the same consideration from the recruitment authorities irrespective of your decision.

**WHAT IF I HAVE QUESTIONS OR PROBLEMS?**

For any questions about the study or any injuries arising from your participation in the study contact the study coordinator at your recruitment site. In case of any emergencies you may contact your doctor at the recruitment centre or the Principal Investigator Dr. Tahir Oshe Umar (Major General) on telephone number 08038249415.

For any questions about your rights as a participant in the study or any complaints about the study, please contact the Chairman of MODRHEC (Dr. Joy Ugwuegede) on +234-802-309-3714 or email: [joyugwuegede@yahoo.com](mailto:joyugwuegede@yahoo.com).
